# Supplementary material for: TCR Sequencing in Mouse Models of Allorecognition Unveils the Features of Directly and Indirectly Activated Clonotypes
Source: Int J Mol Sci. 2023 Jul 28;24(15):12075. doi: 10.3390/ijms241512075 (PMC10418307; doi:10.3390/ijms241512075)
Supplement: Supplementary file 1 [file ijms-24-12075-s001.zip › Supplementary_Material.pdf]

Supplementary Material

TCR sequencing in mouse models of solid organ transplantation unveils the features of allorecognition.

Tereshchenko V., Shevyrev D., Fisher M., Bulygin A., Khantakova J, Sennikov S.
*** Correspondence**: Tereshchenko Valeriy tervp91@gmail.com tereschenko.vp@talantiuspeh.ru

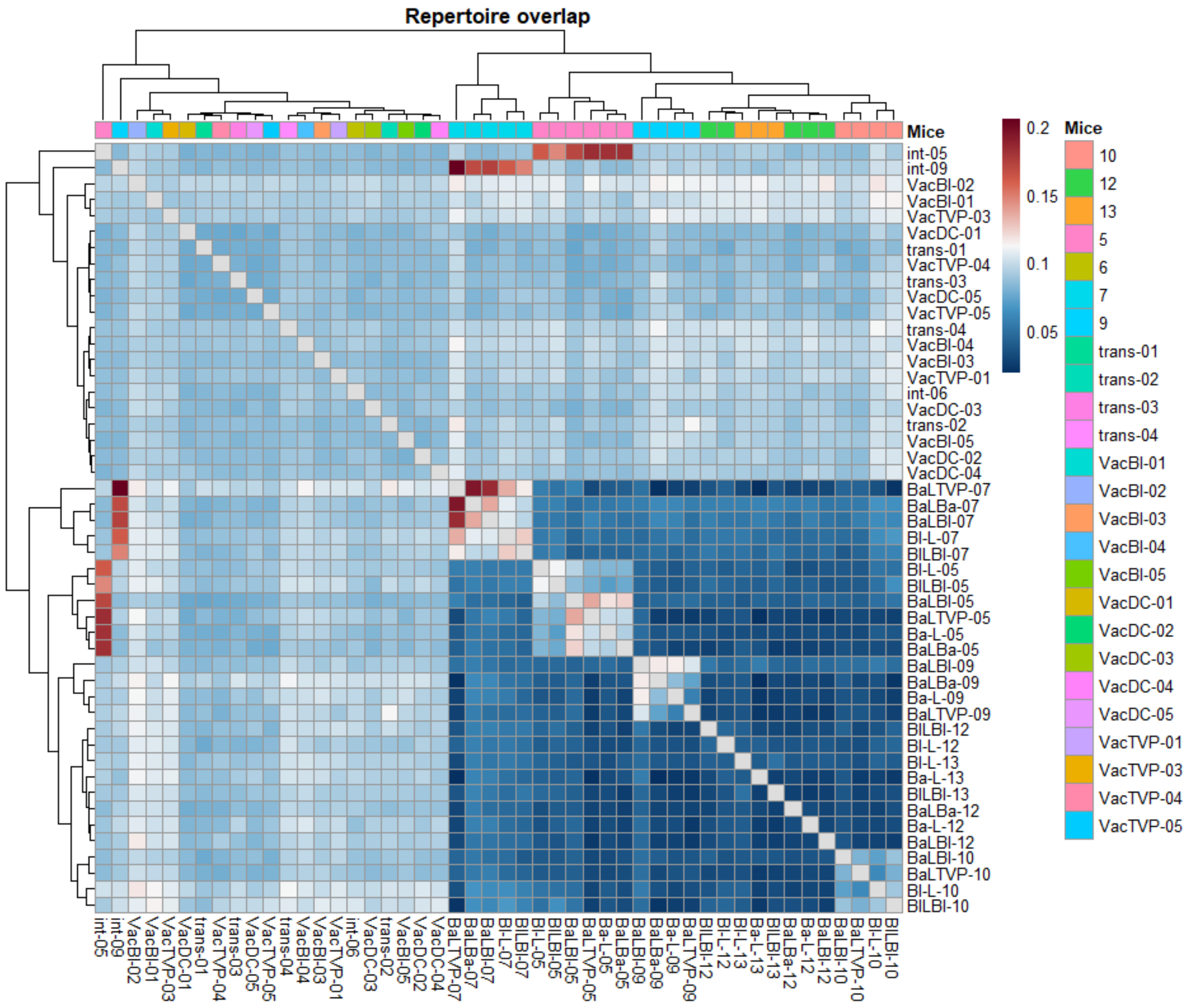

Figure S1. βTCR repertoire overlap of all obtained samples. Samples obtained from the single mouse depicted with the single color. Mice ID on axes. Morisita's similarity index. 0 – complete dissimilarity, 1 – complete similarity

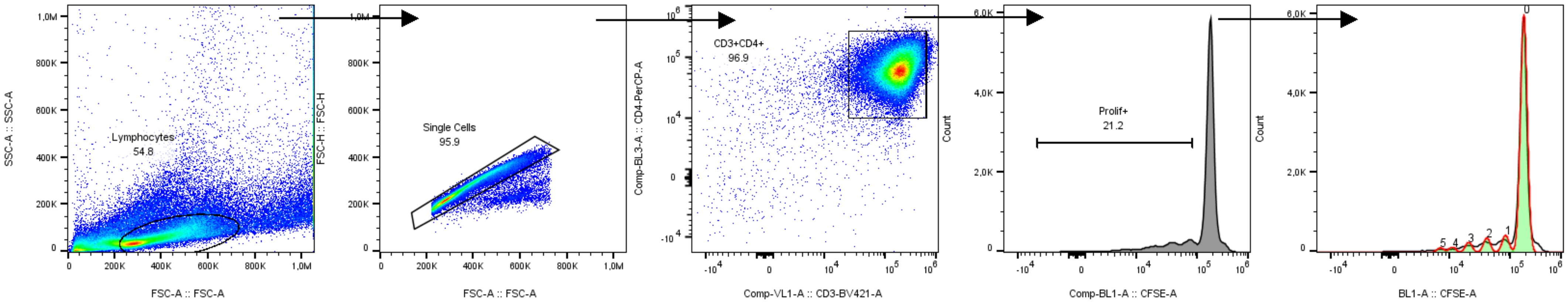

Figure S2. An example of CD4^{+} lymphocytes proliferation in MLC measured by CFSE dilution.

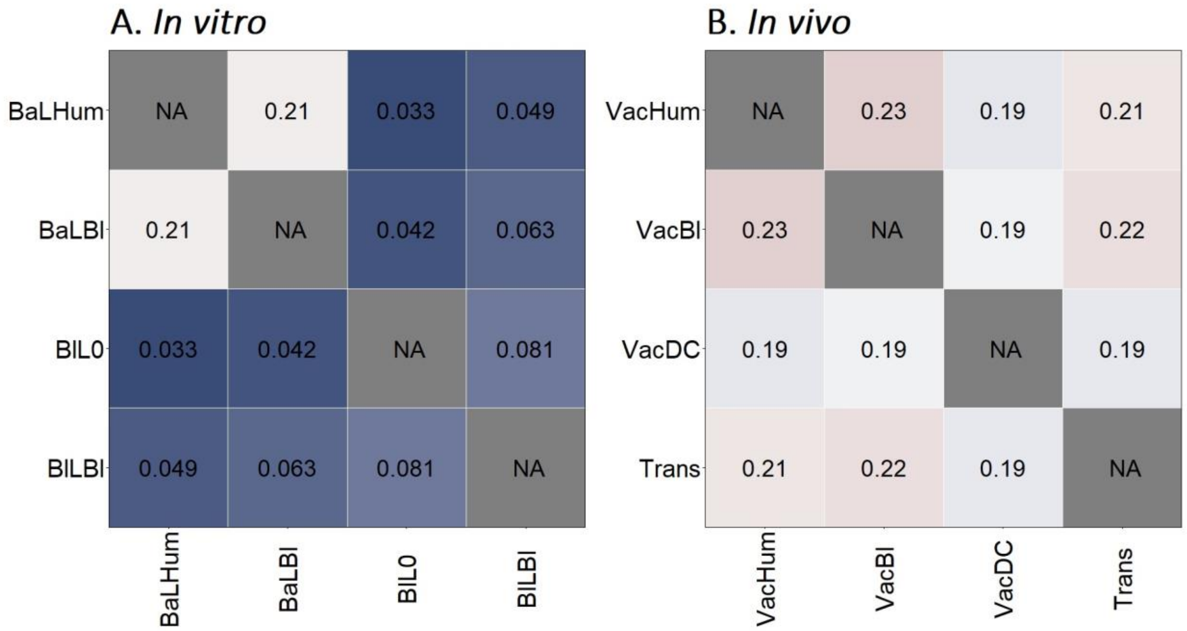

Figure S3. βTCR repertoire overlap of unexpanded *in vitro* and *in vivo* samples combined by stimulation type. Morisita's similarity index. 0 – complete dissimilarity, 1 – complete similarity.

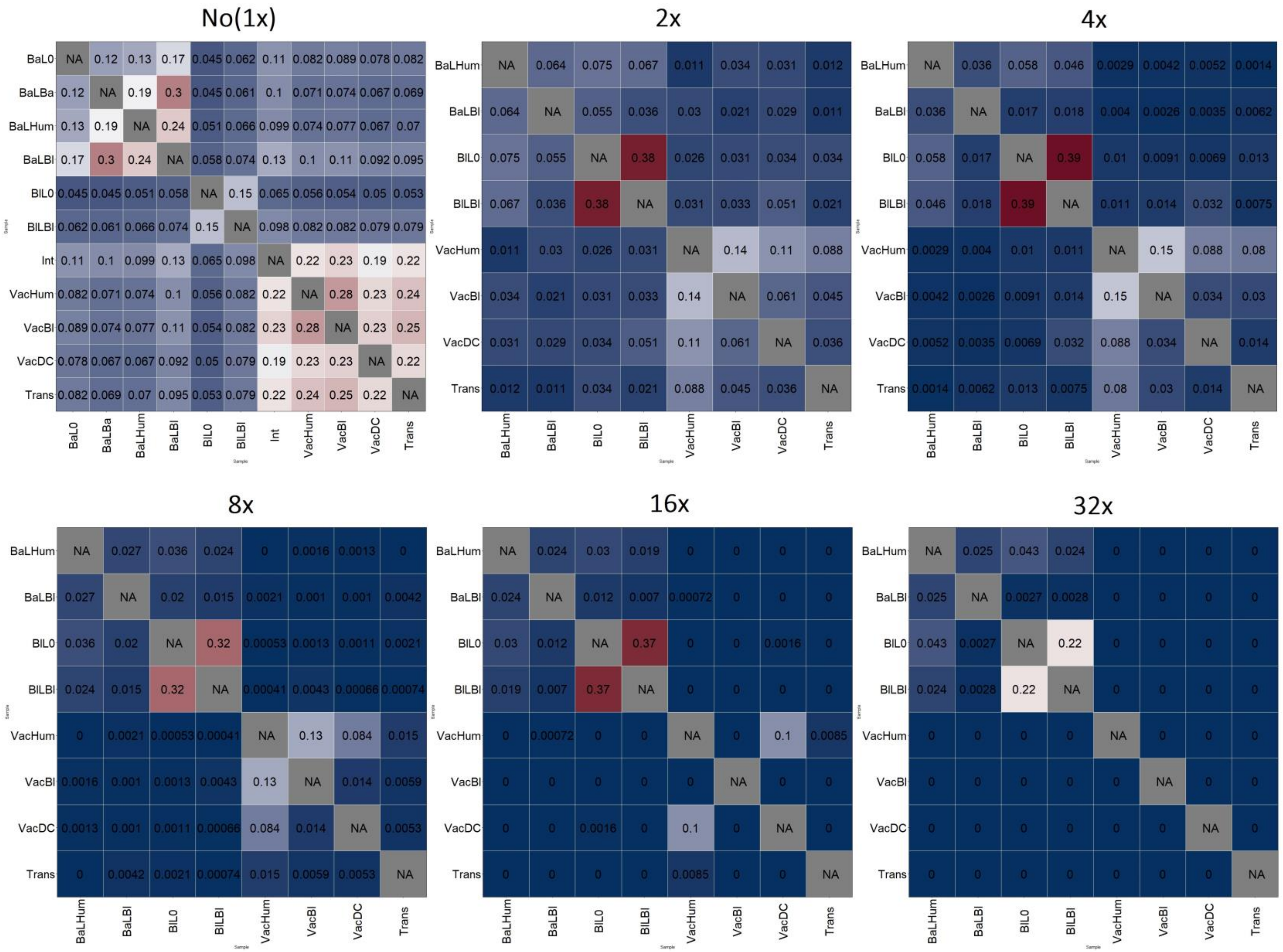

Figure S4. βTCR repertoire overlap of *in vitro* and *in vivo* samples combined by stimulation type. No(1x), 2x, 4x, 8x, 16x, 32x – fold expansion threshold. Morisita's similarity index. 0 – complete dissimilarity, 1 – complete similarity.

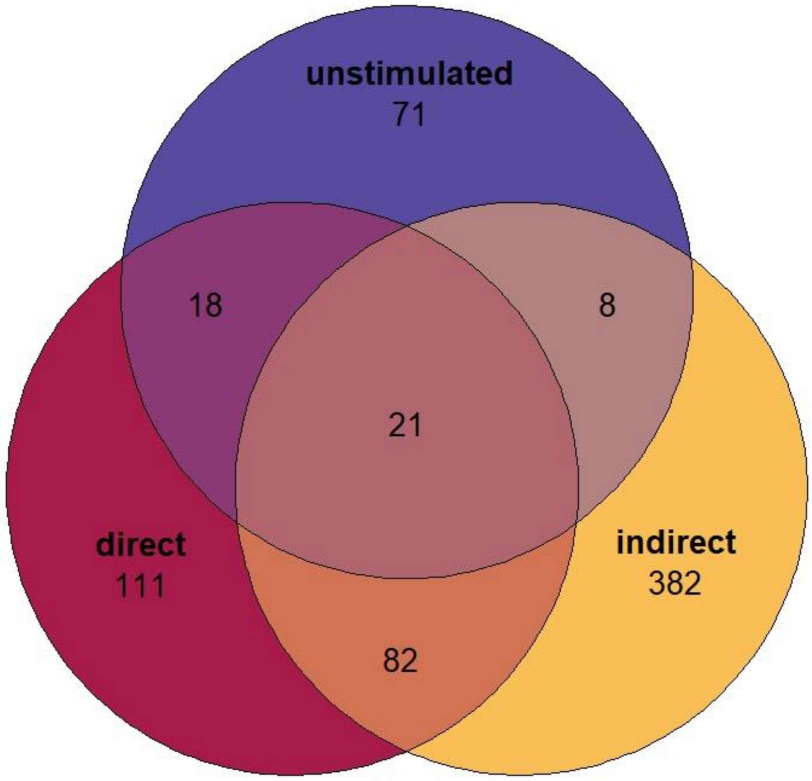

Figure S5. A. An example of $CD4^+$ lymphocytes proliferation in MLC measured by flow cytometry. B. Intersection of GLIPH2 a.a. patterns for directly, indirectly alloreactive and unstimulated repertoires.

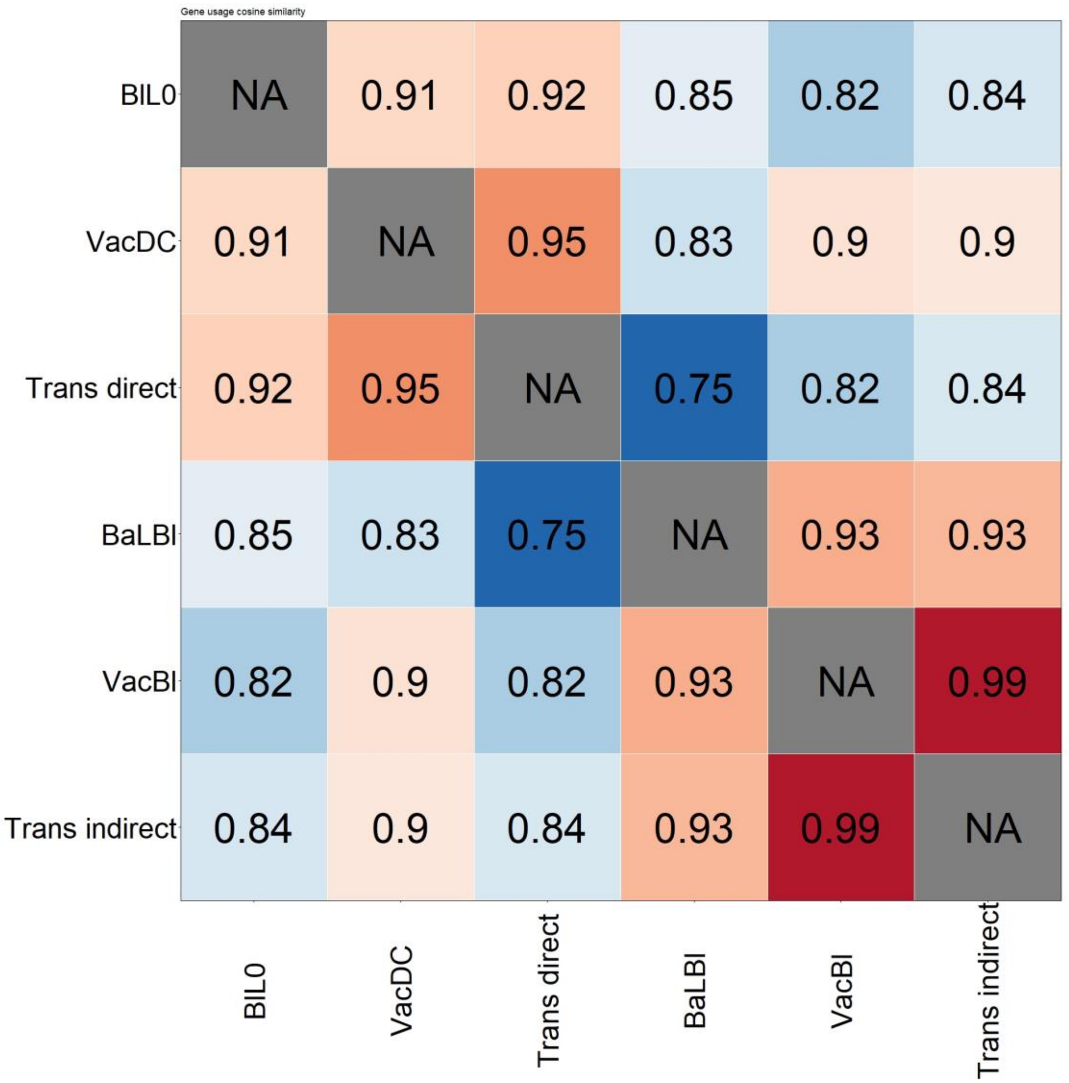

Figure S6. Cosine similarity of V gene usage of clones corresponding to direct (BlL0, VacDC, Trans direct) and indirect (BaLBl, VacBl, Trans indirect) aa patterns. 0 – complete dissimilarity, 1 – complete similarity.
